# Evaluation of the Effect of Anterior Teeth Torque Values on the Space Occupied by Six Anterior Teeth: a Finite Element Analysis

**DOI:** 10.30476/DENTJODS.2021.88649.1346

**Published:** 2022-06

**Authors:** Hosniye Zia Edini, Bahare Fatemipour, Mohammad Mousavi, Hossein Darijani, Mohsen Moeini, Amin Dehghan

**Affiliations:** 1 Dept. of Orthodontics, Oral and Dental Diseases Research Center, Kerman University of Medical Sciences, Kerman, Iran; 2 Dept. of Orthodontics, Zahedan University of Medical Sciences, Zahedan, Iran; 3 Dentofacial Deformities Research Center, Research Institute of Dental Sciences, Dental School, Shahid Beheshti University of Medical Sciences, Tehran, Iran; 4 Dept. of Mechanical Engineering, Shahid Bahonar University of Kerman, Kerman, Iran; 5 Student of Mechanical Engineering, Shahid Bahonar University of Kerman, Kerman, Iran; 6 Student of Mechanical Engineering, Iran University of Science and Technology, Tehran, Iran

**Keywords:** Torque, Orthodontic Space Closures, Finite Element Analyses

## Abstract

**Statement of the Problem:**

Various factors have been introduced to achieve normal occlusion. One of them is anterior teeth torque that has a significant effect on orthodontic treatment outcomes.

**Purpose:**

The aim of this study was to investigate the effect of changes in anterior teeth torque on changes in the space occupied by six anterior teeth by using computer-aided three-dimensional interactive application (CATIA).

**Materials and Method:**

In this experimental finite element study, acrylic teeth with pre-adjusted MBT braces were aligned and three-dimensional (3D) scans were made by 3Dscaner. In the CATIA software program, upper incisors’ torque was changed to -2, -4, -6, +2, +4 and+6 degrees and in the lower incisors to -1, - 3, -5, +3 and+5 degrees; the space was measured at 3 heights of maxillary incisor crowns and at incisal edges of mandibular incisors. Then maxillary incisors were then tapered and the measurements were made again. To evaluate the effect of tooth size, these procedures were carried out on teeth with +17% and -17% magnifications.

**Results:**

The results showed that by increasing anterior torque from 14.7 to 20.7 degrees in maxillary incisors, the space occupied by anterior teeth increased. Maximum changes were at cingulum height with 1.421mm. Reduction in anterior torque from 14.7 to 8.7 degrees resulted in a decrease in this space and maximum changes were observed in the cingulum height with 1.824mm. In the mandibular arch, a 10-degree change in anterior torque resulted in an -.752mm change in the space. Changes in the space occupied by anterior teeth was not significant in tapered and normal teeth in +6 and -6-degree torque (*p* Value= 0.78 and *p* Value=0.83).

**Conclusion:**

By increasing or decreasing the incisors’ torque, the space occupied by anterior teeth increased and decreased, respectively. These changes were less in tapered teeth. Size variations had no significant effect on the results.

## Introduction

Orthodontic treatment aims to achieve dentofacial esthetics and ideal jaw function. One of the most important factors in achieving this goal and resolving the patients’ problems is to achieve proper occlusion. Proper positioning of all the teeth is necessary to form stable and functional occlusion and to position the teeth in a proper relationship with each other and in equilibrium with maxillofacial soft and hard tissues [ 1 - 3 ].

In 1963, Dempster *et al.* [ 1 ] evaluated the inclination of the long axis of teeth in eleven skulls with normal occlusion and reported that all the teeth had an inclination relative to the occlusal plane, which was necessary for achieving the best function.

**Figure 1:**
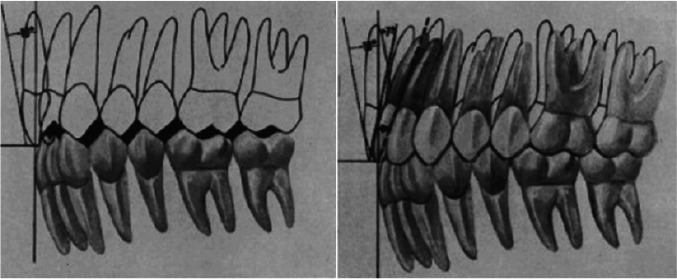
Proper torque of the crown in the anterior results in proper contact points between the upper posterior teeth and their antagonists in the lower jaw, helping establishment of proper posterior occlusion

**Figure 2:**
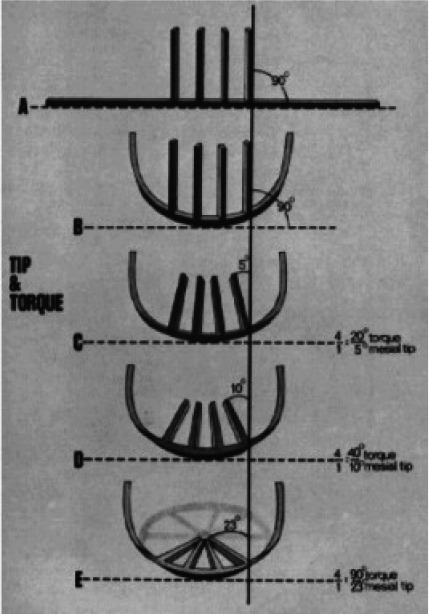
The cartwheel model, with an increase in lingual torque there is a proportional amount of mesial tipping of the roots of anterior teeth, with a relative ratio of 4:1 [ 2

Proper torque of the crown in the anterior region (central and lateral incisors) is one of the most important factors in occlusion; in this context, this torque prevents super eruption of teeth and results in proper contact points between the upper posterior teeth and their antagonists in the lower jaw, helping establish the proper posterior occlusion [ 2 - 4 ] ( Figure 1). In addition, the crown torque clearly affects the overbite and overjet. The torque of the maxillary incisors is particularly important in creating a beautiful smile line, proper anterior guidance, and a stable class I relationship because incisors with inadequate torque (under torque) prevent distal movement of anterior dentition [ 2 - 3 ]. In the cartwheel model of Andrew, a number of vertical wires are soldered on a rectangular arch, as anterior teeth. When the anterior segment of the arch is affected by lingual torque, the vertical wires become convergent so that when the torque is applied at a 90º angle, the wires can be considered as the spokes of a wheel [ 2 ] ( Figure 2).

O’Higgins *et al.* [ 5 ] evaluated the effects of the torque of maxillary incisors on the dental arch space. They used Typodont acrylic resin teeth and natural maxillary incisors with different torques. Then impressions were taken from the set-ups. A reflex microscope was used to measure the arch length from the first incisors on the cast. They concluded that the arch length increased approximately 1mm with a 5-degree increase in the torque of incisors. 

Nanda and Hassel [ 4 ] used mathematical and geometric formulae to quantify the effects of angulation and torque of incisors on the arch length. They concluded that changes in angulation and torque and their reciprocal effects might have had only a minor effect on the arch length; thereby, they did not report any significant changes.

Quantitatively, the effect of torque values on arch length has not been clear yet. In the study of Nanda and Hassel [ 4 ], teeth were converted into small elements and they used mathematical and geometric formulae to quantify the effects of angulation and torque of incisors on the arch length. In order not to complicate the results, the number of elements were few and normal form and morphology of teeth had been changed. They were not as similar as normal teeth. In O’Higgins *et al.*’s study [ 5 ], torque values were not changed in arranged intervals and simulation of clinical situation was made with less effort.

Considering the importance of the torque of tooth crowns in achieving proper occlusion and the limitations of previous studies [ 2 , 4 - 5 ], the present study was conducted to quantify the effects of the torque of anterior teeth on the treatment outcomes, with the use of more accurate techniques, and to evaluate the effects of changes in the torque of anterior teeth on the space occupied by six anterior teeth. 

## Materials and Method

In the present study, three-dimensional (3D) scanning of the samples was prepared from Typodont acrylic resin teeth and analyses with computer-aided three-dimensional interactive application (CATIA) (Dassault systems, France) were used to evaluate the changes in the torque and the form of the tooth crown.

**Figure 3:**
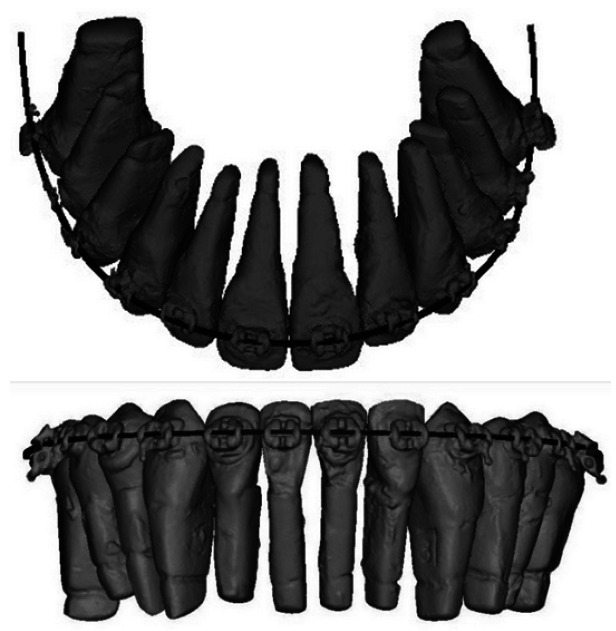
3D scans of upper and lower jaws

First 22-slot pre-adjusted MBT Dentaurum brackets in standard sizes were placed on Typodont acrylic resin teeth. Then elastomeric ligatures (Ortho Technology, USA), were used to place them on a full-thickness prefabricated steel arch wire (SS Ortho Technology, USA), measuring 0.021×0.025 inch. An image was prepared from both the upper and lower arches using a 3D scanner (GOM 2015, Germany, accurate up to 5 µm), which consisted of the points cloud [ 6 ] ( Figure 3).

**Figure 4:**
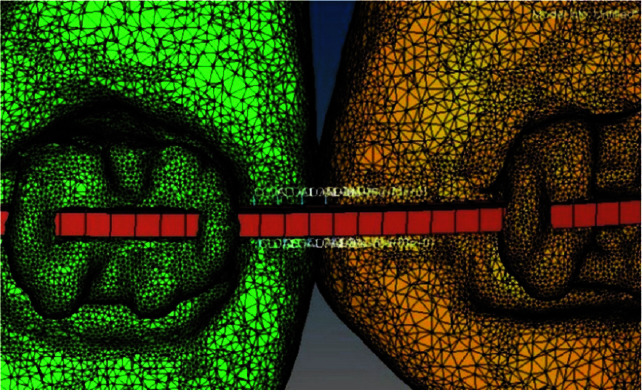
3D model was converted to small elements

**Figure 5:**
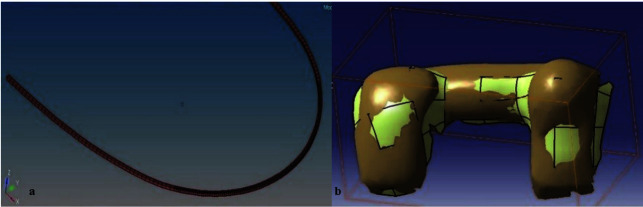
**a:** The point cloud of wire was converted to small elements. For more accurate analysis, the anterior portion of wire consists of smaller elements, **b:** 3D scanning of a bracket; point cloud (brown)

Using the CATIA software program (Dassault systems, France), the points cloud was converted to a smooth surface through scanning, and then the points cloud of the teeth, wires and brackets were converted to small elements ( Figures 4-5).

**Figure 6:**
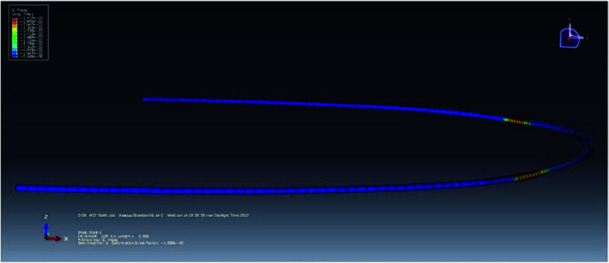
3D scan of a rectangular wire with anterior torque

The teeth were sectioned in the software program and each tooth was saved as separate points cloud. Mechanical properties were added to the wire elements. Based on the mechanical properties and degrees of torque that were favorable in this study, the Abaquscae software program, which is a finite element-based analytical software program, calculated the magnitudes of couple forces that should be applied to achieve the torque values ( Figure 6).

**Figure 7:**
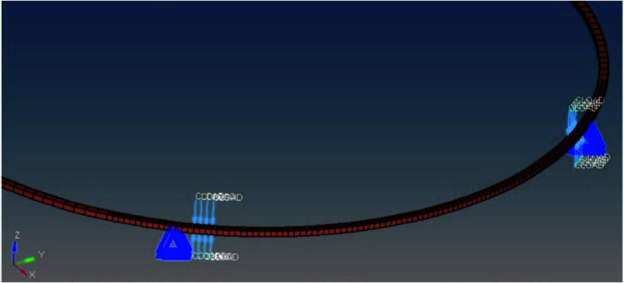
A number of wire elements were considered as fixed points (dark blue) and opposing (couple) forces were applied to the wires (light blue)

Subsequently, a number of wire elements were considered as fixed points and opposing (couple) forces were applied to the wires ( Figure 7). The built-in torque in MBT brackets is 17º; as a result, 25*21 steel arch wire, with a freedom of 2.33º, applies a torque of 14.7º to the incisor teeth [ 7 ]. Torque values applied to the maxilla to evaluate changes in the spaces occupied by six anterior teeth were +2, +4, +6, -2, -4, and -6.

**Figure 8:**
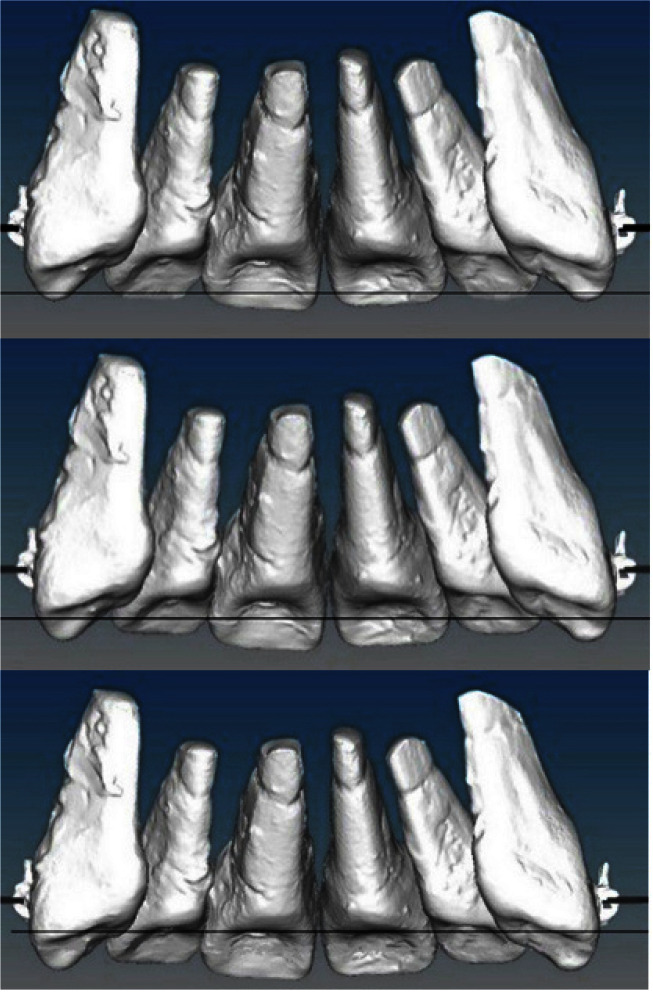
Application of torque. Measurements of changes in the spaces occupied by six anterior teeth were carried out at three heights of the crowns of upper incisors

To make the study more relevant clinically, a decision was made to evaluate changes in the spaces occupied by six anterior teeth with the limits of the line of occlusion. Concerning the differences in the overbite in the normal range of overbite, measurements were carried out at three heights of the crowns of upper incisors. To this end, the distance from the incisal edge to the cingulum was divided into three equal heights using planes that crossed from the distal aspect of the canine on one side to the distal aspect of the canine on the other side; then the space was measured in the planes after application of each torque (Figure 8).

**Figure 9:**
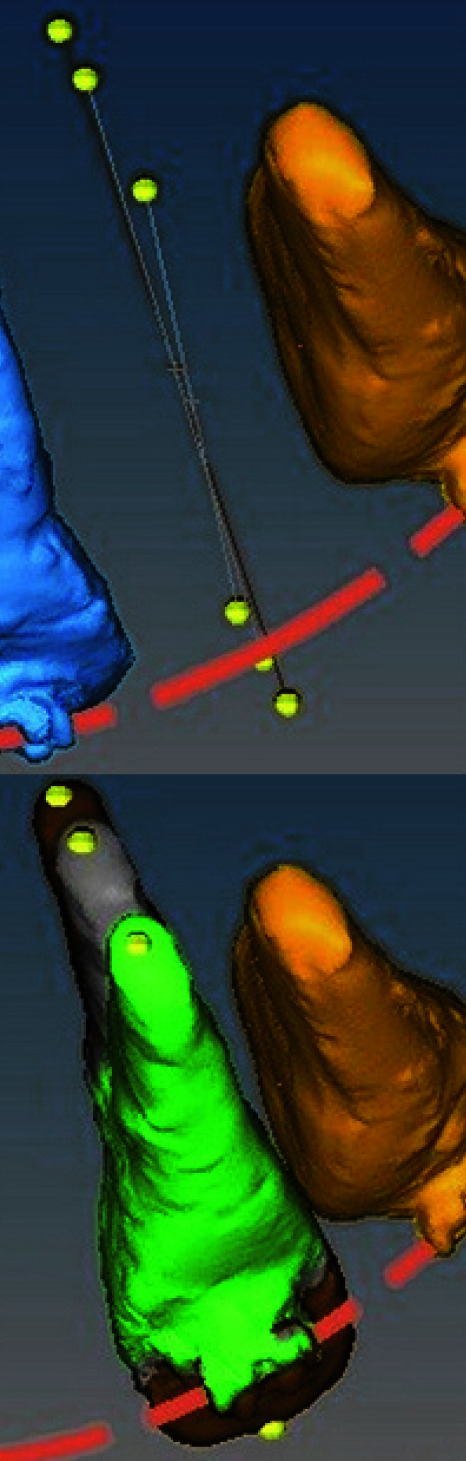
Measurement of changes in the mesiodistal inclination of the teeth with a change in torque

Measurements of changes in the spaces occupied by six anterior teeth in upper jaw in case of increased bite, were carried out at the level of cingulum after the application of +6,0, -6 torque degrees. In addition, the amount of change in the mesiodistal inclination of the teeth was measured with a change in torque (Figure 9). 

**Figure 10:**
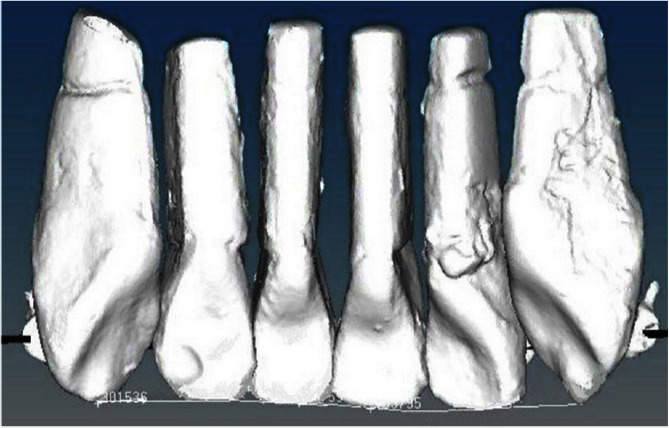
Measurements of changes in the spaces occupied by six anterior teeth in lower jaw at the level of incisal edge

In the lower jaw the torques consisted of -1, -3, -5, +3, and +5, which were applied based on the standard torque values reported in different studies for different bracket systems, and changes in space were measured at the incisal edge [ 8 - 9 ] (Figure 10).

Ovoid shaped teeth have smooth line angles with curved outlines; while triangular shaped teeth have sharp line angles and t are more tapered from incisal to cervical. Therefore, the cervical region of triangular teeth is narrower. In ovoid teeth, connector is located more incisally and contact point is small. In triangular teeth, incisal embrasure is smaller. The CATIA software program was used to reconstruct the tapered shape of crown by changing the size, the location of the connector, and the size of the incisal embrasure. Then again, the procedures for changing the torque and for measuring the space occupied by six anterior teeth were repeated and the results were compared with the values collected from the initial teeth.

**Figure 11:**
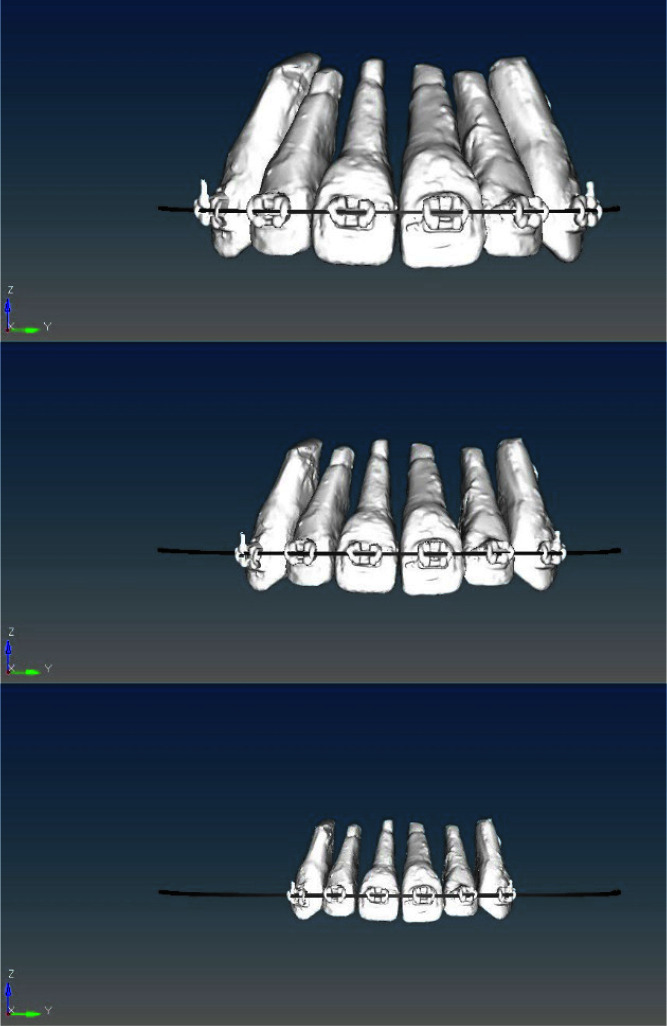
Measurements on the scanned samples of the upper jaw were repeated with 17% of magnification and 17% of decrease in size and the results

Finally, since the maximum and minimum widths of normal maxillary central incisors have been reported to be 9.6 and 7.2 mm, respectively, in order to evaluate the effect of tooth size on changes in space resulting from changes in the arch, the measurements on the scanned samples of the upper jaw were repeated with 17% of magnification and 17% of decrease in size and the results were reported [ 10 - 11 ] (Figure 11).

### Statistical analysis

Data were transferred to Excel and SPSS 18 software programs and classified, summarized, and adjusted using proper methods and presented in the form of tables.

Independent-samples t-test was used to compare changes in the spaces occupied in terms of crown form. Significant differences were defined as a *p*< 0.05.

## Results

**Figure 12:**
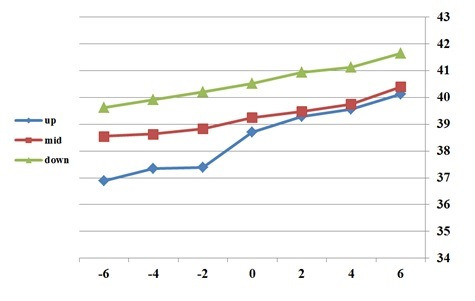
Numeric values of spaces occupied by anterior teeth in different parts of the maxilla at positive and negative values of torque

Tables 1 and 2 present the numeric values of spaces occupied by anterior teeth in different parts of the maxilla at positive and negative values of torque. An important fact is that a 2-degree decrease in tooth torque in the area near cingulum resulted in a 1.33mm decrease in the space occupied by anterior teeth (Table 1,2, Figure 12). 

**Table 1:** Values of spaces occupied by anterior teeth in different parts of the maxilla at positive values of torque

Site of Measurements	0	+2	+4	+6	Difference between 0 to 6
Up	38.705	39.29	39.554	40.126	1.421
Mid	39.236	39.466	39.753	40.398	1.162
Down	40.51	40.931	41.128	41.643	1.133

**Table 2:** Values of spaces occupied by anterior teeth in different parts of the maxilla at negative values of torque

Site of Measurements	0	+2	+4	+6	Difference between 0 to 6
Up	38.705	37.382	37.339	36.881	-1.824
Mid	39.236	38.831	38.63	38.546	-0.69
Down	40.51	40.205	39.92	39.614	-0.896

Based on the data collected in the upper jaw at +6 and -6 torque degrees, the spaces occupied by anterior teeth in the cingulum area in case of increased bite, changed from 32.424 to 30.842mm ( Table 3). The amount of change in the mesiodistal inclination of the teeth measured with a change in torque was measured. It was shown that there was 0.92° of mesial tipping of the root with each 4° increase in the lingual torque of the root.

**Table 3:** Spaces occupied by anterior teeth in the cingulum area and upper third (just beneath cingulum) in the upper jaw at +6 and -6 torque degrees

Torque degree	-6	0	+6
Arch length at cingulum	30.842	31.653	32.424
Arch length beneath cingulum	36.881	38.705	40.126

Based on the results, in the lower jaw, the spaces occupied by the lower teeth at the incisal edge changed 0.752 mm from +5 to -5 degrees ( Table 4).

**Table 4:** Spaces occupied by the lower teeth at the incisal edge

Site of measurement	-5	-3	-1	0	+3	+5	Range of differences between -5 to +5
Up	30.038	30.186	30.343	30.436	30.595	30.79	0.752

In relation to the mean spaces occupied by anterior teeth at three heights of the crowns of triangular-shaped anterior teeth, these values at -6 and +6 torques were 38.562 and 40.481 mm, respectively. In addition, these values at both positive and negative torque values in the triangular-shaped teeth were lower compared to normal teeth; however, there were no significant differences between the means of these changes in mean torque values ( Table 5). 

**Table 5:** Comparison of the mean spaces occupied by anterior teeth at three heights of the crowns of triangular-shaped and normal anterior teeth

Torque value	Tooth shape	Mean	Standard deviation	*p* Value
+6	Normal	40.722	0.98	0.78
	Triangular	40.481	0.92	
-6	Normal	38.347	0.80	0.83
	Triangular	38.526	0.85	

In addition, at +6 and -6 degrees, the spaces occupied by upper anterior teeth in the middle area at 17% magnification changed 1.202 and -0.77 mm, respectively, with changes of 1.06 and -0.58 mm at -17% magnification, respectively ( Table 6).

**Table 6:** Changes of spaces occupiedby upper anterior teeth in the middle area at 17% and -17% magnifications at +6 and -6 degrees of torque

Tooth size	-6	6
Normal	-0.69	1.162
%17 magnification	-0.77	1.202
%-17 magnification	-0.58	1.06

## Discussion

Considering the clinical importance of the torque of anterior teeth in occlusion and introduction of 3D scanners and the high accuracy of this technique, an attempt was made in the present study to evaluate these changes with the use of these highly accurate techniques. The results showed that in upper anterior teeth (central and lateral incisors) with normal anatomy, an increase in torque from 14.7° to 20.7° resulted in an increase in the spaces occupied by six anterior teeth up to 1.42, 1.62 and 1.13 mm, respectively, at three heights from the cingulum to the vicinity of the incisal edge. In addition, with a decrease in torque from 14.7° to 8.7°, the spaces occupied by the anterior teeth decreased 1.82, 0.69, and 0.89 mm, respectively, in the three heights described above. Therefore, the maximum and minimum circumferences were observed near the cingulum at +6 and -6 torque degrees, respectively, with a difference of 3.24 mm between these two. The results of the present study are different from those of a study by Nanda ad Hussel [ 4 ], who quantified the effects of angulation and torque of the incisors on the arch length with the use of mathematical equations. Based on the study above, changes in angulation and torque and their cumulative effects might have a minor effect on arch length. One of the reasons for the discrepancy between the results of these two studies might be the fact that in the study above only geometric measurements were used, which might not be extended to clinical conditions. Furthermore, in order not to complicate the results in Nanda and Hussel study [ 4 ], the numbers of elements were few and normal form and morphology of teeth had been changed or distorted. They were not as similar as normal teeth. In our study, simulation of teeth morphology is almost perfect and geometric calculations by the software are more accurate.

In the present study, a 1.38-mm increase in space was observed for each 6° increase in torque, which was twice that reported by O’Higgins *et al.* [ 5 ], who reported an increase of 0.59‒0.69 mm in arch length with a 5° increase in torque in acrylic resin teeth. This value in natural teeth with a larger size was 0.9‒2.25 mm. since only two bracket states were used to apply torque, it was not possible to evaluate different torque degrees.

To explain changes in tooth circumferences with changes in torque, Andrew’s hypothesis on the relationship between mesiodistal tipping and labiolingual torque of the tooth can be mentioned. Based on this hypothesis, concomitant with an increase in lingual torque in the anterior area of rectangular arch wire, there is a proportional amount of mesial tipping of the roots of anterior teeth, with a relative ratio of 4:1; i.e., there is one degree of mesial tipping of the root and crown divergence with every 4 degrees of increase in the lingual torque of the root [ 2 ].

In the present study, the changes in tipping resulting from changes in the torque were also measured and it was shown that there was 0.92° of mesial tipping of the root with each 4° increase in the lingual torque of the root. These changes in tipping that depend on changes in torque result in the divergence of the tooth crowns and their positioning on a larger arch, leading to in an increase in the circumference of the arch.

In addition, in the study of O'Higgins *et al.* [ 5 ], more changes were reported in the arch circumference in natural teeth, which was attributed to the large size and morphology of natural teeth. A magnification of 17% was used to evaluate the effect of tooth size based on tooth size ranges. At this magnification, at +6 and -6 torques there were 0.04 and 0.08 mm of change, respectively, in spaces relative to normal state; at -17% magnification at -6 and +6 torques, these changes were 0.1 and 0.11 mm, respectively.

In the present study, a change in tooth crown shape to triangular configuration resulted in mean spaces of 40.48 and 38.52 mm, occupied by anterior teeth at three crown heights at +6 and -6 torques, respectively; however, these values in anterior teeth with normal crown anatomy at similar torques were 40.72 and 38.34mm, respectively, which was not significant statistically, consistent with the results reported by O’Higgins *et al.* [ 5 ].

Evaluation of the results of measurements at -1, -3, -5, +3, and +5 torques in the lower jaw showed that a decrease in torque from +5° to -5° resulted in a 0.75-m decrease in the occupied space.

A common phenomenon in the final stages of treatment, especially in extraction cases, is the decreased torque in the crown. Third-order tooth corrections are difficult and time-consuming. Torque correction requires extensive remodeling of bone, which results in some complications [ 11 ]. One of the most important and challenging steps during orthodontic treatment in cases associated with extractions of premolar teeth is the space closure stage. In some of such patients, extra space might be seen in the distal aspect of the lateral tooth during closure of the space in the upper arch with the use of segmental technique, while the canine relationship is class I and no space is present in the lower jaw and no extra overjet is seen. Furthermore, during en-masse space closure, there is space distal to the canine while overjet is zero and the canine relationship is still class II. In the majority of such cases, this problem is only attributed to the presence of Bolton discrepancy. Bolton analysis should be considered during diagnosis, treatment planning, and determination of the prognosis of orthodontic treatment [ 9 , 11 ].

Despite 20‒30% prevalence of anterior Bolton discrepancy [ 12 - 14 ], an important consideration is the fact that such a difference in tooth sizes, which is <2 mm and is achieved through Bolton analysis, is not clinically important [ 15 ]. However, if the torque of the anterior teeth is not properly managed during treatment, the effect of inadequate torque is added to the effect of anterior Bolton discrepancy, resulting in aggravation of problems associated with space closure and achievement of solid occlusion.

For example, according to Araujo *et al.* [ 16 ], the mean anterior Bolton discrepancy in class II subjects is 78.16%. Considering the normal amount of anterior Bolton discrepancy, which is 77.2% [ 17 ], it might be claimed that the amount of discrepancy in the size of teeth in such individuals is approximately 0.4‒0.5 mm. Since such a discrepancy is negligible clinically, it is possible that a sizeable proportion of patients, in which the space discrepancy during treatment is attributed to Bolton discrepancy, have in fact a combination of mild Bolton discrepancy and decreased torque of the anterior teeth.

On the other hand, the results of the present study showed that an increase in overbite resulted in a 1.82-mm decrease in the space occupied by anterior teeth. Therefore, in cases in which an increase in overbite is concomitant with a decrease in the torque in anterior teeth and with possibly Bolton discrepancy, major problems might occur during orthodontic treatment.

In patients with class II div2 malocclusion, there is a combination of increased overbite and incisors with decreased torque. In addition, there is a strong relationship between class II div2 malocclusion and developmental anomalies of teeth, resulting in a 3-fold increase in the incidence of these anomalies in subjects with class II div2 malocclusion compared to the general population. There is a statistically significant decrease in the mesiodistal width of permanent incisors in subjects with this malocclusion, resulting in Bolton discrepancy [ 18 ].

The mean Bolton discrepancy in these subjects has been reported to be 83%. The excess tooth mass in the anterior segment of the mandible or tooth mass deficiency in the anterior segment of the maxilla might even be approximately 2 mm. Therefore, in some of these individuals, association of Bolton discrepancy and decreased torque might give rise to the aggravation of problems in achieving normal occlusion during treatment. Therefore, in these subjects it is necessary to pay attention to the presence and amount of Bolton discrepancy and decreased torque before undertaking treatment in proper treatment planning and by extracting mandibular incisors if necessary [ 17 ].

In these cases, en-masse anterior retraction might exacerbate decreased torque, leads to termination of treatment with class II canine relationship and the absence of overjet. In some of these patients, use of techniques that preserve and correct anterior torque, such retraction of canines separately and use of closing loops with torque for anterior retraction seem necessary for ideal termination of treatment. In addition, use of arch wires with exaggerated curve of spee in growing subjects might be useful [ 19 ].

In cases in which retraction of anterior teeth is carried out by skeletal anchorage (mini screws), usually the line of action of force crosses the lower part of the resistance center of the anterior segment, which results in the loss of torque in anterior teeth. In such cases, the use of indirect anchorage for retraction along with closing loops with torque or long-lever arms [ 20 ] is highly recommended.

In patients with class III malocclusion, usually class III elastics are used during orthodontic treatment. Spena *et al.* [ 21 ] have suggested that anterior brackets in anterior maxilla be placed in reverse so that maxillary incisors would have better inclination after treatment. By assuming a fixed position for the incisal edge, a decrease in torque in upper incisors results in a decrease in the space occupied by six anterior teeth, helping correct class III canine relationship.

In the lower jaw, due to the small number of changes in space, they do not have any clinical effects; however, they might be effective in the relapse of the results of treatment. Based on Schaeffer *et al.* [ 22 ] study on the growth, incisal movement patterns might be seen in three forms with aging and due to the residual growth including an increase in inclination, a decrease in inclination, and no change in inclination. In cases in which fixed and long-term retainers are not used for retention, these changes in inclination lead to isolated tooth rotation and finally to crowding.

Apart from esthetic considerations, based on the hypothesis of Reed and Holdaway [ 23 ], bodily retraction of upper incisors results in a greater decrease in SNA angle, which improves the outcomes from stability points of view. Therefore, stripping of lower teeth is not sufficient for only compensating extra space resulting from a decrease in torque. 

On the other hand, a decrease in torque increases the patient’s bite. If the contact of the incisal edge of anterior teeth is transferred from the lower part of cingulum to a point on the cingulum due to a decrease in torque, the amount of space occupied by six anterior teeth will suddenly decrease up to 6mm ( Table 3), which cannot be compensated by stripping in the lower jaw. 

Therefore, striping is not advisable in extraction cases in the lower jaw except for cases such as the presence of anterior Bolton discrepancy. However, usually in cases in which no extraction is carried out in the lower jaw, stripping is more acceptable by the patients.

## Conclusion

The results of the present study showed that changes in anterior torque affect changes in the space occupied by anterior teeth. The greatest changes were observed in the cingulum area of the anterior teeth. Changes were lower in teeth with triangular crowns. No significant differences were observed in changes, regarding tooth sizes.

## Acknowledgement

The present article was financially supported by Oral and Dental Diseases Research Center, Kerman University of Medical sciences.

## Conflict of Interest:

None declared.
